# RpoN Promotes *Pseudomonas aeruginosa* Survival in the Presence of Tobramycin

**DOI:** 10.3389/fmicb.2017.00839

**Published:** 2017-05-12

**Authors:** Darija Viducic, Keiji Murakami, Takashi Amoh, Tsuneko Ono, Yoichiro Miyake

**Affiliations:** ^1^Department of Oral Microbiology, Institute of Biomedical Sciences, Tokushima University Graduate SchoolTokushima, Japan; ^2^Department of Molecular Microbiology, Institute of Health Biosciences, Tokushima University Graduate SchoolTokushima, Japan

**Keywords:** *Pseudomonas aeruginosa*, RpoN, RpoS, tobramycin, antibiotic tolerance

## Abstract

*Pseudomonas aeruginosa* has developed diverse strategies to respond and adapt to antibiotic stress. Among the factors that modulate survival in the presence of antibiotics, alternative sigma factors play an important role. Here, we demonstrate that the alternative sigma factor RpoN (σ^54^) promotes survival in the presence of tobramycin. The tobramycin-sensitive phenotype of logarithmic phase Δ*rpoN* mutant cells is suppressed by the loss of the alternative sigma factor RpoS. Transcriptional analysis indicated that RpoN positively regulates the expression of RsmA, an RNA-binding protein, in the *P. aeruginosa* stationary growth phase in a nutrient-rich medium. The loss of RpoS led to the upregulation of *gacA* expression in the nutrient-limited medium-grown stationary phase cells. Conversely, in the logarithmic growth phase, the Δ*rpoS* mutant demonstrated lower expression of *gacA*, underscoring a regulatory role of RpoS for GacA. Supplementation of tobramycin to stationary phase Δ*rpoN* mutant cells grown in nutrient-rich medium resulted in decreased expression of *gacA, relA*, and *rpoS* without altering the expression of *rsmA* relative to wild-type PAO1. The observed downregulation of *gacA* and *relA* in the Δ*rpoN* mutant in the presence of tobramycin could be reversed through the mutation of *rpoS* in the Δ*rpoN* mutant background. The tobramycin-tolerant phenotype of the Δ*rpoN*Δ*rpoS* mutant logarithmic phase cells may be associated with the expression of *relA*, which remained unresponsive upon addition of tobramycin. The logarithmic phase Δ*rpoS* and Δ*rpoN*Δ*rpoS* mutant cells demonstrated increased expression of *gacA* in response to tobramycin. Together, these results suggest that a complex regulatory interaction between RpoN, RpoS, the Gac/Rsm pathway, and RelA modulates the *P. aeruginosa* response to tobramycin.

## Introduction

*Pseudomonas aeruginosa* is a Gram-negative pathogen that possesses an extremely high capacity to survive in different environmental niches and poses serious medical risk to immunocompromised patients and patients with cystic fibrosis (CF) (Rau et al., 2010; Silby et al., 2011). One of the major problems is the high resistance of *P. aeruginosa* to a wide range of antibiotics (Lister et al., 2009; Poole, 2011). To overcome the effect of antimicrobials without the expression of a specific resistance mechanism, *P. aeruginosa* employs a strategy known as antibiotic tolerance. This phenomenon allows the adaptation to antibiotic stress through the production of antibiotic-tolerant persister cells. These cells demonstrate the capacity to withstand the effects of elevated antibiotic levels and the ability to persist (Lewis, 2008, 2012). The presence of antibiotic-tolerant cells is the major cause of recurrent infections (Fauvart et al., 2011). Therefore, greater understanding of the molecular mechanism underlying antibiotic tolerance could lead to the development of strategies to prevent recurrent infections caused by *P. aeruginosa*.

The stationary growth phase of *P. aeruginosa*, which produces a substantial amount of cells tolerant to antibiotics (Keren et al., 2004), is characterized by the production of different virulence factors governed by the activation of quorum sensing (QS) systems, which are organized in a hierarchical cascade with the *las* system controlling the expression of the *rhl* system and the *Pseudomonas* quinolone system (PQS) serving as a link between the *las* and *rhl* QS systems (Pesci et al., 1997; McKnight et al., 2000). Activation of the QS system and the corresponding production of virulence factors affects the response of *P. aeruginosa* to antimicrobials (Möker et al., 2010; Que et al., 2013). A number of genes of *P. aeruginosa* including *relA, spoT, dksA*, the alternative sigma factors, RpoS and RpoN, and the signaling molecule PQS, promote the production of antibiotic-tolerant populations (Murakami et al., 2005; Viducic et al., 2006, 2007; Häussler and Becker, 2008; Kayama et al., 2009; Nguyen et al., 2011).

Sigma factors are indispensable for the control of transcription and the regulation of a wide range of genes implicated in diverse functions within the cells, making them important targets for interactions with antimicrobial agents (Kazmierczak et al., 2005). The alternative sigma factor RpoS (σ^s^) is the master regulator of the stationary phase and is involved in the regulation of QS; the QS system positively controls the expression of RpoS (Fujita et al., 1994; Whiteley et al., 2000; Schuster et al., 2004). Another alternative sigma factor, RpoN (σ^54^), is involved in the regulation of nitrogen, motility, mucoidy, and QS (Totten et al., 1990; Heurlier et al., 2003; Thompson et al., 2003; Damron et al., 2012; Sana et al., 2013). Furthermore, we have recently reported that RpoN employs PQS and PqsE to overcome the action of carbapenems (Viducic et al., 2016).

In *P. aeruginosa*, given the roles of RpoN in the regulation of major metabolic pathways, the regulatory control of the translational apparatus, and antibiotic tolerance (Totten et al., 1990; Schulz et al., 2015; Viducic et al., 2016), it is of interest to further our understanding of the mechanism of the RpoN-dependent network in response to aminoglycosides, such as tobramycin. Tolerance to tobramycin has been mainly addressed in context of biofilm-formed cells (Whiteley et al., 2001; Bjarnsholt et al., 2005) however, the key mediators of the response to tobramycin in planktonic cells have not been elucidated. Tobramycin is commonly used in the treatment of *P. aeruginosa* respiratory infections in patients with CF (Ratjen et al., 2009). To kill bacteria, aminoglycosides must bind to the 30S ribosomal subunit and interfere with translation. Furthermore, aminoglycosides induce the insertion of misread proteins into the bacterial cell membrane, affecting membrane integrity and leading to cell death (Davis, 1987; Kohanski et al., 2008). Aminoglycosides can kill both growing and non-growing cells, making them very useful in the treatment of chronic infections (Spoering and Lewis, 2001).

It has long been established that metabolic stress conditions trigger the production of ppGpp, an alarmone of the stringent response that controls a cellular switch leading to translational arrest, modulation of gene expression for the promotion of cell survival, and is an essential trigger of antibiotic tolerance (Cashel et al., 1996; Potrykus and Cashel, 2008; Amato et al., 2014). Because mutation of *rpoN* affects expression of the sigma factor RpoS, which is implicated in the antimicrobial stress response, and is positively regulated by ppGpp (Murakami et al., 2005; Kayama et al., 2009; Battesti et al., 2011), and the existence of σ^54^-dependent activation of *relA* in nitrogen-starved cells (Brown et al., 2014), we have chosen to investigate how the interaction of RpoN with RpoS modulates the response to tobramycin.

Our data demonstrate that RpoN predominantly facilitates the survival to tobramycin in nutrient-rich and nutrient-limited media. The inactivation of *rpoS* in the Δ*rpoN* mutant background in the logarithmic growth phase eliminates the tobramycin-sensitive phenotype of the Δ*rpoN* mutant, suggesting that the RpoS-dependent pathway has an important role in defining the response to tobramycin through the activity of *relA* as well as consequent ppGpp production. Transcriptional analyses demonstrated that in response to tobramycin, the Δ*rpoN* mutant differentially regulates the expression of the *gacA, rsmA, relA*, and *rpoS* genes.

## Materials and methods

### Bacterial strains and culture conditions

The bacterial strains, plasmids, and primers used and generated in this study are shown in Table 1. Bacteria were routinely cultured at 37°C in Luria Bertani medium (LB), AB medium supplemented with 0.2% glucose, 0.2% casamino acids (CAA) (Clark and Maaløe, 1967) and 1 mM L-glutamine for growth of the Δ*rpoN* mutant, or on LB-agar plates supplemented with 10% sucrose when necessary. Vogel-Bonner minimal medium (VBMM) (Vogel and Bonner, 1956) was used in mating experiments. Antibiotics for plasmid selection and propagation were added as required: gentamicin (20 μg/ml) and ampicillin (100 μg/ml) (for *E. coli*), and gentamicin (100 μg/ml) and carbenicillin (400 μg/ml) (for *P. aeruginosa*)

**Table 1:** **Bacterial strains, plasmids, and oligonucleotides used in the study**.

***E. coli***		
DH5α	F−*endA1 hsdR17 supE44 thi-1 recA1 gyrA96 relA1* Δ(*lacZYA-argF*) *U169 deoR* λ(φ80d*lacZ*Δ*M15*)	TakaRa
S17-1 λ*pir*	*pro thi hsdR*^+^ Tp^r^ Sm^r^; chromosome::RP4-2 Tc::Mu-Kan::Tn7/λpir	Simon et al., 1983
***P. aeruginosa***		
PAO1	Wild-type	Stover et al., 2000
PAO1 Δ*rpoN*	PAO1 in-frame deletion of *rpoN*	Viducic et al., 2016
PAO1 Δ*rpoN*Δ*rpoS*	PAO1 in-frame deletion of *rpoN* and *rpoS*	This study
PAO1 Δ*rpoS*	PAO1 in-frame deletion of *rpoS*	Viducic et al., 2017
PAO1 Δ*rpoN*/*rpoN*^+^	Δ*rpoN* mutant carrying a wild-type copy of *rpoN* on pMMB67EH	This study
**Plasmids**		
pEX18Gm	Broad-host-range gene replacement vector; *sacB*, Gm^r^	Hoang et al., 1998
pEX18Gm-Δ*rpoN*	*rpoN* deletion suicide vector	Viducic et al., 2016
pEX18Gm-Δ*rpoS*	*rpoS* deletion suicide vector	Viducic et al., 2017
pMMB67EH	IncQ broad-host-range cloning vector, Ap^r^	Fürste et al., 1986
pMMB67EH-*rpoN*	pMMB67EH containing a functional *rpoN* gene in the opposite orientation to the *tac* promoter, Ap^r^	This study
**Primers for mutant construction and complementation:**		
rpoN-up-F	ATAGAATTCCGATCTCGGTCGGCGACATC	Viducic et al., 2016
rpoN-up-R	ATAGGATCCCTGGAGGTCCAGGGTGGATAG	Viducic et al., 2016
rpoN-down-F	ATAGGATCCGGCATAGCCCCTTCGAGCGAG	Viducic et al., 2016
rpoN-down-R	ATAAAGCTTCTCCGGCAGCTCCCTGGCTA	Viducic et al., 2016
rpoS-up-F	CATTCAGGTCGGTCAAGCTATCCA	Viducic et al., 2017
rpoS-up-R	*TCCGTCACTGTGCCATGTCG*TTATCCCTTG	Viducic et al., 2017
rpoS-down-F	*CGACATGGCACAGTGACGGA*AAACCTTAGA	Viducic et al., 2017
rpoS-down-R	GGAAGTCTGGCCGAACATCACGA	Viducic et al., 2017
rpoN-HindIII-F	CCCAAGCTTGGGAAGTCTACCTGGGGCACGAGT	This study
rpoN-EcoRI-R	CCGGAATTCCGGTCCTTGTTGCCCGTGTGTAAGT	This study
**Primers for qRT-PCR:**		
rsmA-F	TGGGTGTCAAAGGGAACCA	This study
rsmA-R	TGGTAAATTTCCTCCCGGTGTA	This study
relA-F	CCCCAAGGAAAACGGCTATC	This study
relA-R	TGGAGTGGGTACGGATCTGTACT	This study
rpoS-F	CACTTCCTTCTCTCCAAACAACA	Viducic et al., 2016
rpoS-R	AGCTGCGTTGCGTCCAA	Viducic et al., 2016
omlA-F	CGAACTATCAACCAGCTGGTG	Viducic et al., 2016
omlA-R	GCTGTGCTCTTGCAGGTTGTG	Viducic et al., 2016

*^a^Tp^r^, Sm^r^, Ap^r^, and Gm^r^, resistance to trimethoprim, streptomycin, ampicillin, and gentamicin, respectively. Introduced restriction sites are underlined, and the sequences introduced for overlap extension PCR are in italics*.

### Reagents

Tobramycin was purchased from Sigma-Aldrich (St. Louis, MO), and was used at a concentration of 32 μg/ml.

### Antibiotic susceptibility testing

The minimum inhibitory concentration (MIC) of tobramycin was determined using the broth microdilution method as previously described (Miyake et al., 1992; Viducic et al., 2016), with the following modifications: bacterial suspensions were incubated in LB medium at a density of 10^6^ CFU/ml. MICs were determined after 24 h of incubation at 37°C. The MIC was defined as the lowest concentration of antimicrobial agent that completely inhibited the growth of the organism, as detected by the unaided eye.

### Time-kill assays

For the time-kill studies, approximately 10^8^ CFU/ml stationary phase cells were challenged after 16 h of cultivation. Cells were washed once, resuspended in fresh LB or AB medium and then grown with antibiotic in a shaker at 37°C for 24 h. For the logarithmic phase time-kill assay, the cells from overnight culture were diluted in the medium and grown to an OD_595_ of 0.3; at this point, the antibiotic was added, and the experiment was continued for 3 h at 37°C. Samples were collected at several time points, 10-fold serial dilutions were prepared with 0.85% NaCl and 100 μl samples were plated onto LB or AB agar plates in duplicate. Microbial killing was assessed at defined time points by counting colonies and calculating the percent survival relative to untreated cells at time zero. Data were collected from at least three independent experiments. Each experiment included one growth control without addition of tobramycin.

### Generation of mutant strains

Unmarked deletions of *rpoN* and *rpoS* were constructed in *P. aeruginosa* PAO1 (Stover et al., 2000) as described previously (Viducic et al., 2016, 2017) using the pEX18Gm suicide vector, which uses the *sacB*-based counterselection method (Schweizer, 1992; Hoang et al., 1998). A single deletion was first constructed in *rpoN*, which was then used to generate the Δ*rpoN*Δ*rpoS* mutant. The plasmids were transformed into *E. coli* S17-1 λ*pir* (Simon et al., 1983) and conjugated into *P. aeruginosa* PAO1 to generate an in-frame deletion of the genes. The transconjugants carrying the integrated plasmid on the chromosome were selected on LB-agar plates containing 10% (wt/vol) sucrose, and sucrose resistant colonies were screened using colony PCR to identify mutants. The deletion was confirmed by PCR and sequencing.

For complementation studies, a 2.5-kb *Hind*III-*Eco*RI fragment encompassing the *rpoN* gene was amplified by PCR, digested with *Hind*III and *Eco*RI, and the generated fragment was subsequently ligated into *Eco*RI-*Hind*III-digested broad-host range vector pMMB67EH (Fürste et al., 1986). In this construct, the *rpoN* gene conserved its own promoter and Shine-Dalgarno sequence and was inserted in the opposite orientation with respect to the *tac* promoter. This construct was conjugated in the Δ*rpoN* mutant.

### RNA isolation and quantitative real-time PCR (qRT-PCR) analysis

For stationary phase RNA isolation, the strains were grown overnight in 10 ml of LB or AB medium at 37°C for 16 h, and then the cultures were washed and resuspended in 10 ml of LB or AB medium. RNA was isolated at a time point before the addition of tobramycin and at 24 h following the growth in the presence of tobramycin. For RNA isolation from the logarithmic phase cells, overnight cultures were used to inoculate 10-ml subcultures in LB to an OD_595_ of 0.01. Cultures were incubated at 37°C to an OD_595_ of 0.3; at this point a sample was taken for RNA isolation. Then, tobramycin was added to the culture, and cultures were incubated for an additional 3 h; cells were then harvested for RNA isolation. Total RNA was isolated from *P. aeruginosa* using RNeasy Miniprep Kit according to the manufacturer's protocol (Qiagen). Total RNA was on column DNase- treated using RQ1 DNase (Promega) and was used as a template in PCR to assess the presence of contaminating DNA. cDNA was generated from 1 μg of DNase-treated RNA using the Transcriptor First Strand cDNA Synthesis Kit (Roche Diagnostics) according to the manufacturer's instructions. qRT-PCR reactions were carried out in a StepOnePlus Real-Time PCR Systems (Thermo Fisher Scientific) using the Fast SYBR® Green Master Mix (Thermo Fisher Scientific), according to the specification of the supplier. To correct for the differences in the amount of starting material, a constitutively expressed *omlA* was used as a reference gene (Ochsner et al., 1999). The oligonucleotide primers used to detect the expression of each gene of interest are listed in Table 1. At least three technical replicates were performed for each cDNA sample analyzed.

### Growth assay

To assess the growth of the wild-type PAO1, the Δ*rpoN*, Δ*rpoS*, Δ*rpoN*Δ*rpoS*, and Δ*rpoN*/*rpoN*^+^ mutants, overnight grown cultures were washed and used to inoculate 10-ml subcultures in LB medium or AB medium to an OD_595_ of 0.01. The absorbance at 595 nm was monitored every 2 h for 12 h, with an additional measurement at 24 h.

### Statistical analysis

The data were statistically analyzed using Student's *t*-test (two-tailed two-sample assuming equal variances) using GraphPad Prism7 software. Definition of statistical significance is *P* < 0.05.

## Results

### Δ*rpoN* mutants are sensitive to tobramycin exposure

The capacity of *P. aeruginosa* to persist in the presence of antimicrobial agents without acquiring resistance mechanisms is attributed to the production of specialized antibiotic-tolerant cells (Keren et al., 2004). One of the characteristics of antibiotic-tolerant cells is that their mechanism of survival usually does not reflect MIC values, which remain unchanged, and allows them to grow in the presence of a high antibiotic concentration (Brauner et al., 2016). To address whether the deletion of *rpoN* and loss of *rpoS* in the Δ*rpoN* mutant background affects the MIC values of tobramycin, MIC determinations were performed. The MIC values of tobramycin for wild-type PAO1 and the Δ*rpoN*, Δ*rpoS*, and Δ*rpoN*Δ*rpoS* mutants were 1 μg/ml.

**Figure 1:**
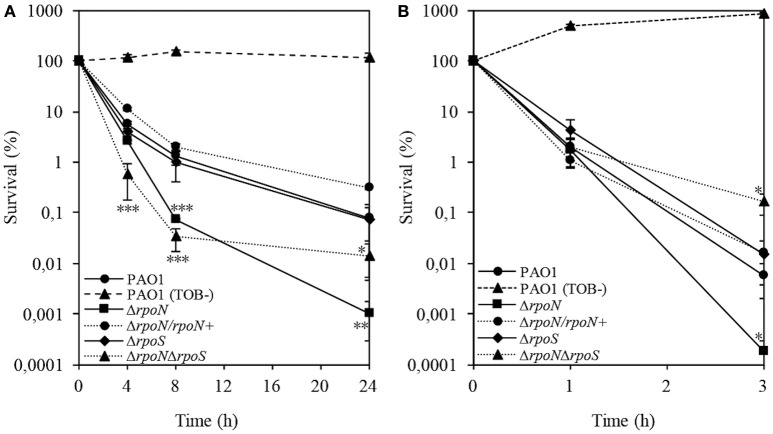
**Time-dependent killing assay of stationary-phase (A)** and logarithmic phase **(B)** wild-type PAO1 and the Δ*rpoN*, Δ*rpoS*, Δ*rpoN*Δ*rpoS*, and Δ*rpoN*/*rpoN*^+^ mutants treated with 32 μg/ml tobramycin grown in LB. Wild-type PAO1 was grown without tobramycin and served as a growth control. Percent survival at the indicated time points was calculated by dividing the number of CFU/ml after antibiotic treatment by the CFU/ml before addition of tobramycin. The experiment was performed in triplicate. Error bars indicate SDs. *P* ≤ 0.05 (^*^), ≤0.01 (^**^), or ≤0.001(^***^) vs. wild type.

The role of RpoN in conferring carbapenem and fluoroquinolone tolerance (Viducic et al., 2007) prompted us to investigate a potential role of RpoN in the interaction with another class of antibiotics, such as aminoglycosides, which target the translational machinery. To address this question, we performed killing assays for stationary phase wild-type PAO1 and the Δ*rpoN* mutant grown in the LB medium in the presence of tobramycin at 32 μg/ml, which is a concentration corresponding to 32 × the MIC. The Δ*rpoN* mutant stationary phase cells exhibited a significant decrease in viability compared to wild-type PAO1, suggesting that in the stationary phase, RpoN promotes survival in the presence of tobramycin (Figure 1A). To confirm that the tobramycin-sensitive phenotype was due to the loss of *rpoN*, we complemented the Δ*rpoN* mutant by introduction of plasmid pMMB-*rpoN*, encoding a wild-type copy of *rpoN* gene. The complemented strain, Δ*rpoN*/*rpoN*^+^, demonstrated a wild-type response to tobramycin, suggesting that the tobramycin-sensitive phenotype was due to the loss of *rpoN* and not due to a secondary mutation.

Previous studies on RpoN have suggested that QS systems and RpoS are negatively regulated by RpoN (Heurlier et al., 2003; Kayama et al., 2009; Viducic et al., 2016). Given the protective role of RpoS in the presence of aminoglycosides (Baharoglu et al., 2013; Wang et al., 2014), we sought to determine whether the response to tobramycin in the Δ*rpoN* mutant could be modulated through RpoS. To assess the role of RpoS in tolerance to tobramycin, we constructed Δ*rpoS* and Δ*rpoN*Δ*rpoS* mutants and performed a killing assay for stationary phase cells in the presence of tobramycin at 32 μg/ml. The level of survival of the Δ*rpoS* mutant in the presence of tobramycin was comparable to that observed for wild-type PAO1. The survival rate of the Δ*rpoN*Δ*rpoS* mutant resembled that observed for the Δ*rpoN* mutant; however, at the 24-h time-point, the Δ*rpoN*Δ*rpoS* mutant showed an increase in survival relative to the Δ*rpoN* mutant, partially overcoming the RpoN-dependent response to tobramycin (Figure 1A). Having confirmed the role of RpoN in response to tobramycin in stationary phase cells, it was of interest to assess how inactivation of *rpoN* affects the response to tobramycin in logarithmic phase cells. To explore this, the cells were grown to an OD_595_ of 0.3 and treated with tobramycin at 32 μg/ml. As demonstrated in Figure 1B, the Δ*rpoN* mutant demonstrated a stark decrease in viability following exposure to tobramycin. These results confirmed that irrespective of the growth phase, the response to tobramycin is RpoN-dependent. In contrast, the Δ*rpoS* mutant demonstrated insignificantly higher survival in comparison to wild-type PAO1 (Figure 1B). The Δ*rpoN*Δ*rpoS* mutant completely abolished the effect of tobramycin from the 1-h time-point throughout the remainder of the experimental timeframe and demonstrated increased survival relative to that of wild-type PAO1 (Figure 1B). Furthermore, when the Δ*rpoN* mutant was complemented with the wild-type copy of *rpoN*, the survival rate to tobramycin resembled that of wild-type PAO1 (Figure 1B).

**Figure 2:**
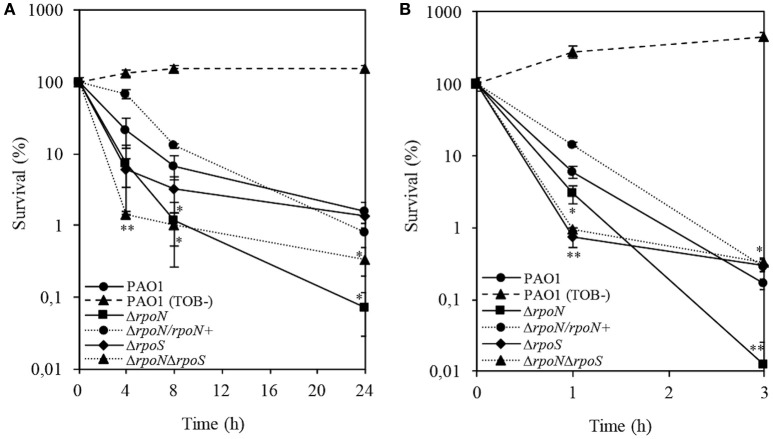
**Time-dependent killing assay of stationary-phase (A)** and logarithmic phase **(B)** wild-type PAO1 and the Δ*rpoN*, Δ*rpoS*, Δ*rpoN*Δ*rpoS*, and Δ*rpoN*/*rpoN*^+^ mutants treated with 32 μg/ml tobramycin grown in a defined minimal (AB) medium supplemented with 0.2% glucose and 0.2% CAA. Wild-type PAO1 was grown without tobramycin and served as a growth control. Percentage survival at the indicated time points was calculated by dividing the number of CFU/ml after antibiotic treatment by the CFU/ml before addition of tobramycin. The experiment was performed in triplicate. Error bars indicate SDs. *P* ≤ 0.05 (^*^), ≤0.01 (^**^) vs. wild type.

Metabolic flexibility and rapid adaptation of *P. aeruginosa* to diverse nutrient-limited conditions often correlates with a decrease in susceptibility to antibiotics (Poole, 2012). Given that RpoN has been implicated in the acquisition of nitrogen and amino acid metabolism (Totten et al., 1990) and that the stress response in *P. aeruginosa* is attributed to RpoS (Suh et al., 1999), we decided to focus subsequent work on investigating tobramycin tolerance by modulating the metabolic status of the cells. To define the importance of nutrient-limited conditions in tobramycin tolerance, we performed killing assays with stationary phase cells in a defined minimal (AB) medium supplemented with 0.2% glucose and 0.2% CAA. The results of the killing assays demonstrated an overall increase in survival to tobramycin in all strains; however, nutrient-limited conditions failed to restore wild-type tolerance in the Δ*rpoN* mutant. The complemented Δ*rpoN* mutant produced wild-type survival in the presence of tobramycin (Figure 2A). Growth in AB minimal medium failed to abolish the tobramycin-sensitive phenotype of the logarithmic phase Δ*rpoN* mutant cells, and the logarithmic phase Δ*rpoS* and Δ*rpoN*Δ*rpoS* mutant cells demonstrated a rapid decrease in survival after 1 h of treatment with tobramycin, which was followed by a delay in killing up to the 3-h time-point. The complemented Δ*rpoN* mutant demonstrated wild-type survival in response to tobramycin (Figure 2B).

**Figure 3:**
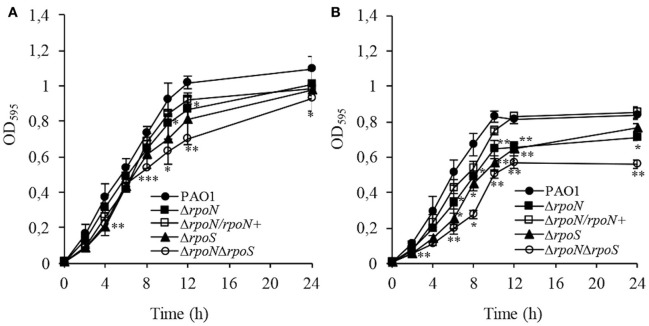
**Growth rate analysis for wild-type PAO1, Δ***rpoN*** mutant, Δ***rpoS*** mutant, Δ***rpoN***Δ***rpoS*** mutant, and Δ***rpoN***/***rpoN***^**+**^ mutant grown in the LB medium (A)** and AB minimal medium supplemented with 0.2% glucose and 0.2% CAA **(B)** at 37°C. Growth curves showing the absorbance at 595 nm plotted over time. Error bars indicate SDs. *P* ≤ 0.05 (^*^), ≤0.01 (^**^), or ≤0.001(^***^) vs. wild type.

Growth rate analysis of the mutants in LB medium indicated that the Δ*rpoN* mutant grew slightly slower than the wild-type PAO1, whereas the Δ*rpoN*Δ*rpoS* mutant displayed a more pronounced growth defect (Figure 3A). Growth in the defined AB minimal medium resulted in significantly delayed growth for the Δ*rpoN*Δ*rpoS* mutant and to a lesser extent for the Δ*rpoN* and Δ*rpoS* mutants (Figure 3B). The complemented strain, Δ*rpoN*/*rpoN*^+^, restored the growth defect of the Δ*rpoN* mutant (Figures 3A,B).

Taken together, these data support the following key findings: (i) RpoN is important for *P*. *aeruginosa* interaction with tobramycin during both the stationary and logarithmic growth phases; (ii) the logarithmic phase response of RpoN to tobramycin is mediated through a mechanism that involves RpoS; and (iii) the observed slow growth correlates with the Δ*rpoN*Δ*rpoS* mutant being recalcitrant to killing by tobramycin.

### Tobramycin differentially affects the expression of *gacA, rsmA, relA*, and *rpoS* depending on the metabolic status of the cells

Based on our time-kill assays, which suggested the interaction of RpoN with tobramycin via RpoS, we further focused our attention on a search for genes that might interfere with the mechanism of action related to aminoglycosides and define the pathway for tobramycin tolerance in the Δ*rpoN* mutant. To modulate external signals into an adaptive response, *P. aeruginosa* employs two-component systems consisting of a sensor kinase that responds to specific signals by modifying the phosphorylated state of a cognate response regulator (Gao et al., 2007; Goodman et al., 2009). Post-transcriptional regulation is one of the mechanisms used by bacteria to adapt to environmental conditions and is regulated by RNA binding proteins that control the translation of target mRNAs (Romeo et al., 2013). GacS/GacA, a two-component system, controls the expression of small trans-acting regulatory RNAs, RsmY, and RsmZ, which interact with RsmA, an RNA-binding protein. RsmA/CsrA binds to multiple sites of the 5′-untranslated region (5′-UTR) close to the Shine-Dalgarno sequence, preventing ribosomal binding, which mediates the post-transcriptional control of genes involved in a number of physiological pathways (Baker et al., 2002; Burrowes et al., 2006; Brencic et al., 2009; Romeo et al., 2013). Taking into account that aminoglycosides act by impairing the integrity of the inner cell membrane through the incorporation of misread proteins, that they must bind the 30S ribosome subunit to interfere with translation to kill bacteria (Davis, 1987; Kohanski et al., 2008) and that RsmA is one of the targets for ribosome interaction and competes with the 30S ribosomal subunit (Baker et al., 2002), we anticipated that the Gac/Rsm pathway might be important for the tobramycin-stress response.

**Figure 4:**
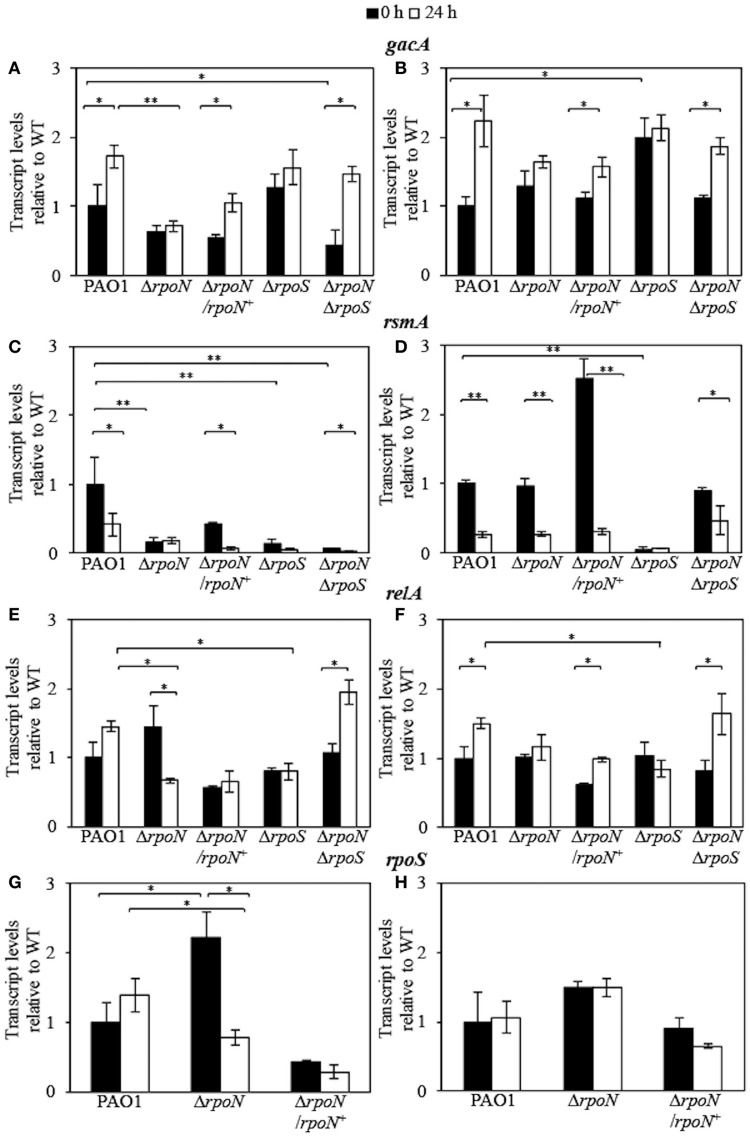
**Expression of ***gacA***, ***rsmA***, ***relA***, and ***rpoS*** genes in stationary phase wild-type PAO1 and the Δ***rpoN***, Δ***rpoS***, Δ***rpoN***Δ***rpoS***, and Δ***rpoN***/***rpoN***^**+**^ mutants grown in the LB medium (A,C,E,G)** and in AB minimal medium **(B,D,F,H)**. The *gacA, rsmA, relA*, and *rpoS* transcript levels were measured by qRT-PCR, were normalized to *omlA* expression, and the levels are expressed relative to the wild-type PAO1 at time = 0 h. The time points at which the cells were sampled for transcriptional analysis were *t* = 0 h and *t* = 24 h after the addition of 32 μg/ml tobramycin, as indicated. All results are the average of at least three independent experiments, and the error bars represent SDs. *P* ≤ 0.05 (^*^), ≤0.01 (^**^).

To determine if tobramycin induces changes in the expression of genes in the Gac/Rsm pathway, we investigated the expression of *gacA* and *rsmA* in wild-type PAO1 and the mutant strains grown to stationary phase in LB and AB media. For all transcriptional analyses using qRT-PCR, we used the samples from the 0 and 24-h time-point of the killing assays. In LB medium, the most prominent difference was observed in the Δ*rpoN*Δ*rpoS* mutant with a 2.2-fold decrease in *gacA* expression relative to wild-type PAO1 (Figure 4A). Whereas *gacA* levels in the Δ*rpoN* and Δ*rpoS* mutants remained unaltered after tobramycin exposure, the Δ*rpoN* mutant still demonstrated a 2.4-fold lower expression relative to wild-type PAO1. The Δ*rpoN*Δ*rpoS* mutant and wild-type PAO1 produced a significant 3.2- and 1.7-fold increase in *gacA* levels after tobramycin exposure, respectively (Figure 4A). The expression of *gacA* in AB medium in the Δ*rpoN* and Δ*rpoN*Δ*rpoS* mutants was comparable to wild-type levels; however, the Δ*rpoS* mutant demonstrated significantly higher levels of *gacA* than wild-type PAO1 (Figure 4B). The addition of tobramycin induced a positive effect on expression of *gacA* in wild-type PAO1 and the Δ*rpoN*Δ*rpoS* mutant, resulting in 2.2- and 1.7-fold increases, respectively (Figure 4B), without significantly affecting *gacA* expression in the Δ*rpoN* and Δ*rpoS* mutants. In contrast to the modest change of *gacA* expression observed in LB medium, the Δ*rpoN*, Δ*rpoS*, and Δ*rpoN*Δ*rpoS* mutants demonstrated a 6.2-, 7.6-, and 14.5-fold lower expression of *rsmA*, respectively, relative to wild-type PAO1 (Figure 4C). The addition of tobramycin to stationary phase cells in LB medium decreased the *rsmA* levels in wild-type PAO1 and the Δ*rpoN*Δ*rpoS* mutant by 2.6- and 1.8-fold, respectively, without significantly altering the levels of *rsmA* in the Δ*rpoN* and Δ*rpoS* mutants (Figure 4C). In AB medium, wild-type PAO1 and the Δ*rpoN* and Δ*rpoN*Δ*rpoS* mutants displayed no difference in *rsmA* expression, and tobramycin addition negatively affected the expression of *rsmA* in these strains (Figure 4D). The Δ*rpoS* mutant demonstrated significantly lower expression of *rsmA* in comparison to wild-type PAO1, and the addition of tobramycin did not alter *rsmA* expression in the Δ*rpoS* mutant (Figure 4D).

To address the correlation between the metabolic status of the cells produced by different growth media on an increase in the levels of ppGpp, a regulator of the stringent response synthesized through the activity of RelA (Cashel et al., 1996), we examined the transcriptional levels of *relA* in wild-type PAO1 and the mutant strains. In LB medium, the highest effect was observed in the Δ*rpoN* mutant, which produced a modest 1.45-fold increase in *relA* expression relative to wild-type PAO1 (Figure 4E). The addition of tobramycin led to a significant 2.2-fold decrease in *relA* level in the Δ*rpoN* mutant and a 1.8-fold increase in *relA* level in the Δ*rpoN*Δ*rpoS* mutant while the Δ*rpoS* mutant demonstrated reduced *relA* expression relative to wild-type PAO1 (Figure 4E). The levels of *relA* expression remained similar in all strains in AB medium; however, the addition of tobramycin stimulated a 2-fold increase in *relA* expression in the Δ*rpoN*Δ*rpoS* mutant, with a similar upregulation observed in wild-type PAO1 (Figure 4F). The level of *relA* expression in the Δ*rpoN* and Δ*rpoS* mutants remained unchanged in AB medium irrespective of tobramycin addition, however, the Δ*rpoS* mutant demonstrated significantly lower expression of *relA* in comparison to wild-type PAO1 (Figure 4F).

The RpoS-dependent role in coordinating stationary phase survival (Suh et al., 1999) and the positive effect of *relA*-mediated ppGpp production on *rpoS* expression (Battesti et al., 2011) prompted us to investigate the transcriptional levels of *rpoS*. In LB medium, the expression of *rpoS* was increased in the Δ*rpoN* mutant relative to wild-type PAO1 (Figure 4G). The addition of tobramycin to stationary phase Δ*rpoN* mutant cells in LB provoked a downregulation of *rpoS* expression (Figure 4G). In AB medium, no significant difference in *rpoS* expression was observed in the Δ*rpoN* mutant relative to wild-type PAO1 (Figure 4H).

**Figure 5:**
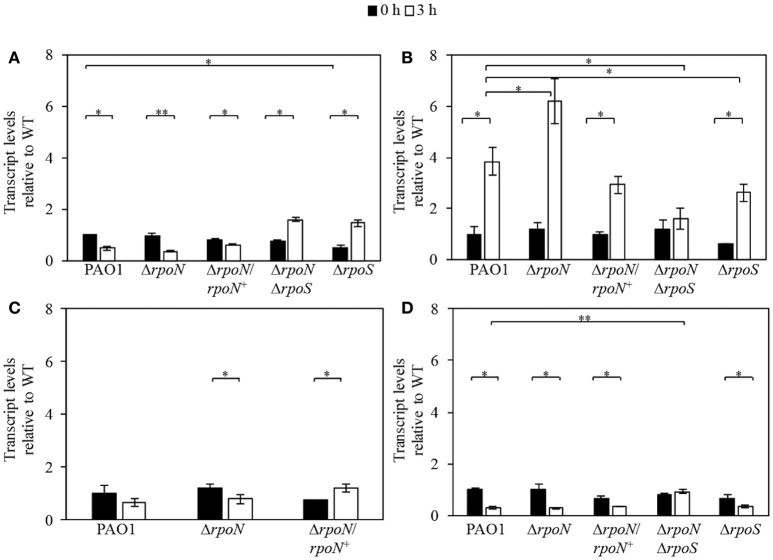
**Expression of ***gacA*** (A)**, *rsmA* **(B)**, *rpoS* **(C)**, and *relA* **(D)** in logarithmic phase wild-type PAO1 and the Δ*rpoN*, Δ*rpoS*, Δ*rpoN*Δ*rpoS*, and Δ*rpoN*/*rpoN*^+^ mutants grown in the LB medium in the presence of 32 μg/ml tobramycin. The transcript levels were measured by qRT-PCR, were normalized to *omlA* expression, and are expressed relative to wild-type PAO1 at time = 0 h. The time points at which the cells were sampled for transcriptional analysis were *t* = 0 h and *t* = 3 h after the addition of tobramycin, as indicated. All results are the average of at least three independent experiments, and the error bars represent SDs. *P* ≤ 0.05 (^*^), ≤0.01 (^**^).

To obtain insight into the underlying molecular mechanism of tobramycin tolerance of the logarithmic phase cells grown in the nutrient-rich LB medium, we performed a transcriptional analysis of *gacA, rsmA, rpoS*, and *relA* expression in wild-type PAO1 and the Δ*rpoN*, Δ*rpoS*, Δ*rpoN*Δ*rpoS*, and Δ*rpoN*/*rpoN*^+^ mutants at time point *t* = 0 h and time point *t* = 3 h after tobramycin addition. The transcriptional analysis revealed significantly lower expression of *gacA* in the Δ*rpoS* mutant relative to wild-type PAO1 (Figure 5A). Whereas tobramycin addition led to a decrease in *gacA* levels in wild-type PAO1 and the Δ*rpoN* mutant, it produced a significant effect in the opposite direction in the Δ*rpoS* and Δ*rpoN*Δ*rpoS* mutants by increasing the transcription of *gacA* by 2.8- and 2.1-fold, respectively (Figure 5A). Based on *rsmA* transcriptional expression, no significant difference was observed between wild-type PAO1 and the Δ*rpoN*, Δ*rpoS*, and Δ*rpoN*Δ*rpoS* mutants and the addition of tobramycin led to a significant increase in *rsmA* levels by 3.8-, 5.3-, and 4.3-fold in wild-type PAO1, the Δ*rpoN* mutant, and the Δ*rpoS* mutant, respectively (Figure 5B). Deletion of *rpoN* did not affect *rpoS* transcriptional levels in the logarithmic phase cells; however, tobramycin induced a decrease in *rpoS* levels in the Δ*rpoN* mutant (Figure 5C).

Wild-type PAO1 and the Δ*rpoN*, Δ*rpoS*, and Δ*rpoN*Δ*rpoS* mutants demonstrated no significant differences in *relA* expression (Figure 5D). However, a prominent downregulation of *relA* expression by tobramycin in wild-type PAO1 and the Δ*rpoN* and Δ*rpoS* mutants by 3.6-, 3.8-, and 1.9- fold, respectively, was evident (Figure 5D). Conversely, in the Δ*rpoN*Δ*rpoS* mutant, the levels of *relA* remained unaltered upon tobramycin addition (Figure 5D). Complementation of the Δ*rpoN* mutant with the wild-type *rpoN* gene demonstrated that the changes in the expression of *gacA, rsmA, relA*, and *rpoS* genes could be attributed to the loss of RpoN.

Taken together, the transcriptional analysis results led to several important conclusions: (i) depending on the nutritional status of the cells, RpoN responds to tobramycin by modulating the expression of *gacA, rsmA, relA*, and *rpoS*; (ii) depending on the growth phase and nutritional status of the cells, the expression of *rsmA* and *gacA* is regulated through a RpoS-dependent pathway; (iii) RpoS modulates *relA* expression in response to tobramycin; (iv) the loss of both RpoN and RpoS in logarithmic phase cells results in *relA* expression that is unresponsive to tobramycin, leading to the increased survival; and (v) RpoN likely affects the survival to tobramycin challenge in the logarithmic phase through a pathway integrally linked to RpoS.

## Discussion

The ability of *P. aeruginosa* to promptly acquire the response to antibiotic-induced stress conditions through its complex regulatory networks is crucial for survival (Morita et al., 2014). The sigma factor RpoN is involved in nitrogen metabolism, carbon assimilation, nutrient transport, motility, mucoidy, and QS regulation (Potvin et al., 2008). Taking into account that the role of RpoN in the tobramycin stress response is defined by its close regulatory association with the stringent response network and by its importance in the regulation of genes involved in the translation apparatus (Brown et al., 2014; Schulz et al., 2015), we were interested in furthering our understanding of the network linking RpoN with the tobramycin stress response. In addition, given that RpoN is involved in the regulation of RpoS (Kayama et al., 2009; Viducic et al., 2016), we aimed to address whether the observed interaction with RpoS is involved in the tobramycin stress response. Due to the significant impact of the metabolic status of the cells on the response to antimicrobial agents (Poole, 2012), our interest was further directed toward investigating how metabolic alterations affect tobramycin stress-response.

**Figure 6:**
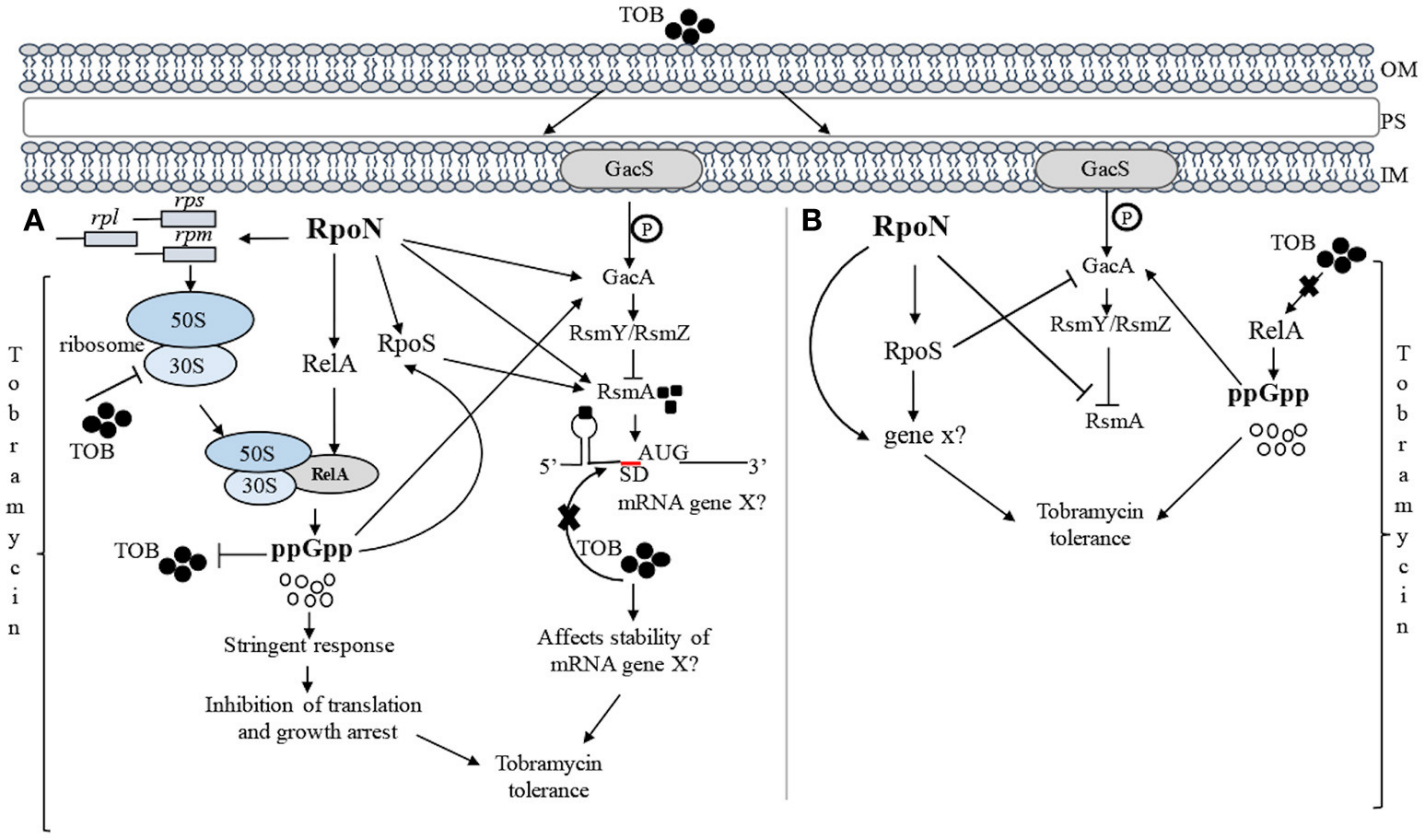
**Proposed model for the involvement of RpoN in the regulation of tobramycin tolerance in the stationary growth (A)** and logarithmic phase growth **(B)** in nutrient-rich medium. **(A)** RpoN stimulates the expression of the genes involved in the translation apparatus and positively affects the synthesis of ribosome components. Upon exposure to tobramycin, RpoN counteracts the negative effects of tobramycin on translation by increasing the expression of *relA*, which in turn produces the effector of the stringent response, ppGpp. ppGpp promotes survival in the presence of tobramycin by inducing translational inactivity and affecting the growth rate. The translational inactivity produced by ppGpp blocks tobramycin from exerting its effect on the ribosome and consequently leads to tobramycin tolerance. In addition, RpoN positively affects the expression of *rpoS* in the presence of tobramycin and employs RsmA to overcome the effect of tobramycin, likely by increasing the mRNA stability of a gene potentially involved in tobramycin tolerance. RpoN increases the expression of *gacA* in response to tobramycin, which probably occurs through a ppGpp-dependent pathway. **(B)** During the logarithmic phase of growth, RpoN employs the RpoS-dependent pathway, which in turn activates additional stress-response genes promoting the survival to tobramycin. RpoN profoundly downregulates the expression of *rsmA* in response to tobramycin. Furthermore, RpoS negatively affects the expression of *gacA* in the presence of tobramycin. The mutant deficient in *rpoN* and *rpoS* demonstrates (i) the loss of tobramycin-mediated inhibition of *relA* expression, (ii) a consequent increase in ppGpp production, and (iii) suggests ppGpp-dependent upregulation of *gacA*, which together lead to tobramycin tolerance. The detailed regulation network is described in the Discussion. OM, outer membrane; PS, periplasmic space; IM, inner membrane; TOB, tobramycin; → represents positive control; ⊥ represents negative control.

In this study, we have demonstrated that RpoN promotes *P. aeruginosa* survival in the presence of tobramycin. The loss of RpoN in the logarithmic growth phase could be alleviated through the inactivation of RpoS, highlighting the importance of interaction between RpoN and RpoS in the tobramycin stress response in the logarithmic growth phase. These observations prompted us to examine the network affected as a consequence of the *rpoN* and *rpoS* inactivation and determine how it correlates with the response to tobramycin. On the basis of our observations, we propose a model of an RpoN-dependent network for interaction with tobramycin (Figure 6). Taking into account that tobramycin requires translation to exert its bactericidal activity, we were interested in examining the regulators that are closely associated with translation. Our results demonstrated that during the stationary growth phase under nutrient replete conditions, RpoN exerts substantial positive control over RsmA. As RsmA is a posttranscriptional regulatory protein, which binds to the 5′-UTR of a mRNA close to the Shine–Dalgarno region and directly blocks the translation of the mRNA (Brencic et al., 2009), this observation suggested that RpoN integrates the RsmA regulatory pathway to modulate tobramycin-mediated changes in cell metabolism. While RpoN does not alter the expression of *gacA* during growth under nutrient replete conditions, the inability of the Δ*rpoN* mutant to increase *gacA* expression in response to tobramycin (the Δ*rpoN* mutant demonstrates a 2.4-fold decrease in *gacA* expression) suggested that RpoN requires GacA to adequately respond to tobramycin.

Furthermore, our transcriptional analysis demonstrated that the loss of RpoS in the Δ*rpoN* mutant background provoked significant alteration in the expression of *gacA* in the absence or presence of tobramycin in stationary phase cells. The support for RpoS-dependent expression of *gacA* comes from the observations that under nutrient-limited conditions and during logarithmic growth phase in LB medium, RpoS affects the expression of *gacA*. Furthermore, our results demonstrated pronounced RpoS-mediated control of *rsmA* expression. Given our results, and the previously published observations on the regulatory link between RsmA and RpoS in *P. aeruginosa* showing that RpoS positively affects the expression of *rsmA* (Schulz et al., 2015; Stacey and Pritchett, 2016), our observations further demonstrate that RpoN likely modulates the expression of *rsmA* through an RpoS-dependent regulatory network. In addition, RpoN might employ RsmA to increase mRNA stability of the gene targeted by tobramycin due to the fact that RsmA may also contribute to positive regulation of mRNA (Romeo et al., 2013; Yakhnin et al., 2013). Phenotypic association of the RsmA- and RpoN-network in biofilm formation, interaction with antimicrobial agents, and regulation of T6SS (Burrowes et al., 2006; Mulcahy et al., 2008; Brencic and Lory, 2009; Sana et al., 2013) further strengthens the complex relationship between these regulators.

One possibility for the importance of RpoS in the RpoN network is that RpoN affects tobramycin tolerance through modulation of *rpoS* expression. This postulation is in accordance with previous observations suggesting that RpoS is an important target for interaction with aminoglycosides (Baharoglu et al., 2013; Wang et al., 2014). Our transcriptional results suggested that RpoN employs RpoS to alleviate the effects of tobramycin in the stationary and logarithmic growth phases. RpoS has a dominant effect on gene expression in the stationary growth phase, controlling approximately 14% of the *P. aeruginosa* genome and exerting a positive regulation over genes involved in the regulation of chemotaxis, two-component regulatory systems, QS, alginate synthesis, and the RpoS-activated *katA* and *lecA* genes (Schuster et al., 2004). Considering the close regulatory association of RpoN with the sensor kinase KinB in the regulation of the genes involved in alginate synthesis, QS, virulence factor production, and carbohydrate metabolism (Damron et al., 2012), there is a substantial overlap between RpoN- and RpoS-dependent gene expression. This overlap underscores the importance of the interaction between RpoN and RpoS in response to diverse environmental stimuli, including antibiotic stress.

In addition, our killing assays demonstrated that RpoS was important in mediating the tobramycin response in the logarithmic cells grown in the minimal medium, while no significant role for RpoS was observed in the stationary phase or logarithmic phase cells grown in LB medium. In contrast with our observations, the importance of RpoS in the response to aminoglycoside-induced stress has been demonstrated in *E. coli* (Wang et al., 2014). Furthermore, RpoS protects *E. coli* and *V*. *cholerae* from subinhibitory concentrations of tobramycin-induced SOS (Baharoglu et al., 2013). In our studies the cells were treated with a high concentration of antibiotic, corresponding to 32 × MIC, suggesting that the RpoS-dependent response can be distinguished only when using lower concentration of tobramycin. In addition to antibiotic concentration, the response to antibiotics may be affected by the growth conditions, growth medium, or the strains used in the study. Furthermore, there appears to be a considerable difference in the expression of genes involved in cell division, cell wall synthesis, as well as adaptation and protection, between the Δ*rpoS* mutant of *P. aeruginosa* and *E. coli* (Schuster et al., 2004). As proposed by Schuster et al., in *P. aeruginosa*, these genes comprise a minor fraction of the genes induced by RpoS, and because they play a vital role in the response to a variety of stresses, this observation may aid in explaining the less sensitive antibiotic stress phenotype of the Δ*rpoS P. aeruginosa* mutant relative to the *E. coli* Δ*rpoS* mutant.

We anticipated that another target for interaction with RpoN might be the *relA* gene, which is responsible for production of the stringent response effector molecule, ppGpp, and is essential for survival of the cells (Cashel et al., 1996). The nutrient status of the cells is an important signal in modulating the intracellular levels of ppGpp by directly activating the expression of *relA*, and it has been demonstrated that the nitrogen stress response integrates ppGpp-mediated changes to adapt to low nitrogen availability (Brown et al., 2014). The observed increase in *relA* transcriptional expression in stationary phase Δ*rpoN*Δ*rpoS* mutant cells in response to tobramycin, irrespective of the nutrient conditions, underscores the importance of ppGpp in tobramycin tolerance. In addition, RpoS affects *relA* expression in response to tobramycin. Activation of the stringent response results in a reduction of ribosomes synthesis due to downregulation of ribosomal RNA (Potrykus and Cashel, 2008). These restrictive conditions impede the action of tobramycin and allow the cells deprived of the alternative sigma factors RpoN and RpoS to antagonize the effect of tobramycin via a mechanism dependent on the *relA* gene.

The present study found that there was no influence of *relA* on the response to tobramycin in logarithmic phase Δ*rpoN* mutant cells as determined by transcriptional analysis, but this study did demonstrate that *relA*-dependent ppGpp production alleviated the effect of tobramycin in the Δ*rpoN*Δ*rpoS* mutant. Consistent with this finding, the inability of the Δ*rpoN* mutant to fine tune the stringent response during tobramycin treatment correlated with a decrease in survival to tobramycin. The impact of RpoN on *relA* expression during the stationary growth phase in the presence of tobramycin led us to postulate, and further to confirm through the observations by Schulz et al., that the role of RpoN in tobramycin tolerance stems from its close association with the ribosome. The evidence of the complex regulatory network interconnected with RpoN was demonstrated using ChIP-seq in combination with a motif scan, in which RpoN was found to regulate 53 genes involved in translation, post-translational modification, and degradation (e.g., genes that belong to the *rpl, rps*, and *rpm* ribosomal clusters (Schulz et al., 2015).

These observations and the findings of this study prompted us to postulate that the prompt response to tobramycin requires RpoN to enhance the expression of *rsmA* and maintain stability of its expression. In this scenario, RpoN acts by employing RsmA to positively affect the mRNA of the target genes by likely involvement in ribosome synthesis and by promoting ribosome-dependent *relA* expression to mount the stringent response, which together subsequently affects survival to tobramycin. While the observations reached in the study by Schulz et al., provide an explanation for the tobramycin-sensitive phenotype of the Δ*rpoN* mutant during the stationary growth phase, in the logarithmic growth phase, RpoN interacts with RpoS and with additional stress-response genes to promote translational inactivity to overcome the effects of tobramycin. This explanation is supported by the data showing no difference in *relA* expression between the Δ*rpoN* mutant and the wild type. Interestingly, the Δ*rpoN* mutant demonstrated significantly higher expression of *rsmA* in the logarithmic phase cells in response to tobramycin, underscoring the importance of interaction between RpoN and RsmA in modulating the response to tobramycin. In support of this observation, Schulz et al. have demonstrated that RpoN controls a number of non-coding RNAs, suggesting that RpoN might employ non-coding RNAs to interact with RsmA to promote the cellular reaction to tobramycin.

It has been demonstrated that ppGpp affects the expression of *csrA*, a homolog of RsmA in *E. coli*, through positive effects on *csrB*/*C* non-coding RNAs, which antagonize CsrA. Furthermore, CsrA directly controls *relA* suggesting that CsrA is engaged in fine-tuning the stringent response (Edwards et al., 2011). In *Pseudomonas fluorescens* CHA0, GacA is involved in the regulation of ppGpp and during nutrient-restricted conditions ppGpp activates the Gac/Rsm pathway (Takeuchi et al., 2012). Concordant with these observations, increased *relA* levels in logarithmic phase cells of the Δ*rpoN*Δ*rpoS* mutant and consequent ppGpp production, suggest the existence of a potential ppGpp-dependent upregulation of *gacA* in the presence of tobramycin.

RpoN-dependent control of aminoglycoside tolerance has previously been reported in *Pseudomonas fluorescens* SBW25 (Jones et al., 2007). Increased susceptibility of the Δ*rpoN* mutant to tobramycin could be attributed to the alterations in the expression of RpoN-controlled flagellar genes, which have recently been implicated in aminoglycoside tolerance (Shan et al., 2015).

Given the increasing evidence supporting the association of ppGpp with tolerance to antibiotics in *P*. *aeruginosa* (Viducic et al., 2006; Khakimova et al., 2013), what we can deduce from the observations of this study is that based on the growth phase and nutritional status of the cells, RpoN cooperates with the RelA-dependent ppGpp production pathway through its regulatory control of the translation apparatus, with RpoS and with the RsmA-dependent pathway to integrate the network required for survival in the presence of tobramycin. Future studies will be required to delineate the RpoN-mediated tobramycin tolerance pathway in order to advance the understanding of the effects of RpoN on cellular functions.

## Author contributions

Conceived and designed the study: DV, KM, TO, and YM; Designed the experiments: DV and KM; performed the experiments: DV, KM, and TA; Analyzed the data: DV, KM, TA, TO, and YM; Wrote the paper: DV and YM.

### Conflict of interest statement

The authors declare that the research was conducted in the absence of any commercial or financial relationships that could be construed as a potential conflict of interest.
